# Methionine Supplementation Alleviates the Germ Cell Apoptosis Increased by Maternal Caffeine Intake in a *C. elegans* Model

**DOI:** 10.3390/nu16060894

**Published:** 2024-03-20

**Authors:** Hyemin Min, Juhae Kim, Mijin Lee, Sangwon Kang, Yhong-Hee Shim

**Affiliations:** Department of Bioscience and Biotechnology, Konkuk University, Seoul 05029, Republic of Korea; mintmin@konkuk.ac.kr (H.M.); fdsa@konkuk.ac.kr (J.K.); miranda12@konkuk.ac.kr (M.L.); cjstlakstl@konkuk.ac.kr (S.K.)

**Keywords:** caffeine, methionine, germ cell apoptosis, yolk protein (vitellogenesis), *C. elegans*

## Abstract

Caffeine (1,3,7-trimethylxanthine) is a widely consumed bioactive substance worldwide. Our recent study showed that a reduction in both reproduction and yolk protein production (vitellogenesis) caused by caffeine intake were improved by vitamin B12 supplementation, which is an essential co-factor in methionine metabolism. In the current study, we investigated the role of methionine in the reproduction of caffeine-ingested animals (CIAs). We assessed the effect of methionine metabolism on CIAs and found that caffeine intake decreased both methionine levels and essential enzymes related to the methionine cycle. Furthermore, we found that the caffeine-induced impairment of methionine metabolism decreased vitellogenesis and increased germ cell apoptosis in an LIN-35/RB-dependent manner. Interestingly, the increased germ cell apoptosis was restored to normal levels by methionine supplementation in CIAs. These results indicate that methionine supplementation plays a beneficial role in germ cell health and offspring development by regulating vitellogenesis.

## 1. Introduction

Caffeine is the most popularly consumed psychoactive stimulant in a variety of foods such as tea, coffee, soda, and some drugs [1]. Its neurological effects are well known from *in vivo* studies of caffeine consumption. It acts as an antagonist of A1 and A2 adenosine receptors; affects the central nervous system; increases adrenaline, dopamine, and noradrenaline; alters fat and carbohydrate metabolism by mobilizing blood circulation and intracellular calcium stores; and stimulates lipolysis in humans [2,3,4]. In addition, 400 mg of caffeine a day appears to be generally safe, enhances physical performance, and confers health benefits, with improvement of chronic liver disease, Type 2 diabetes, Parkinson’s disease, and Alzheimer’s disease [5,6,7]. However, there are growing concerns about caffeine consumption in pregnant women, owing to the increased risk of pregnancy-related adverse effects, including low birth weight and pregnancy loss [8,9]. However, studies on the effects of maternal caffeine intake are limited.

**Figure 1:**
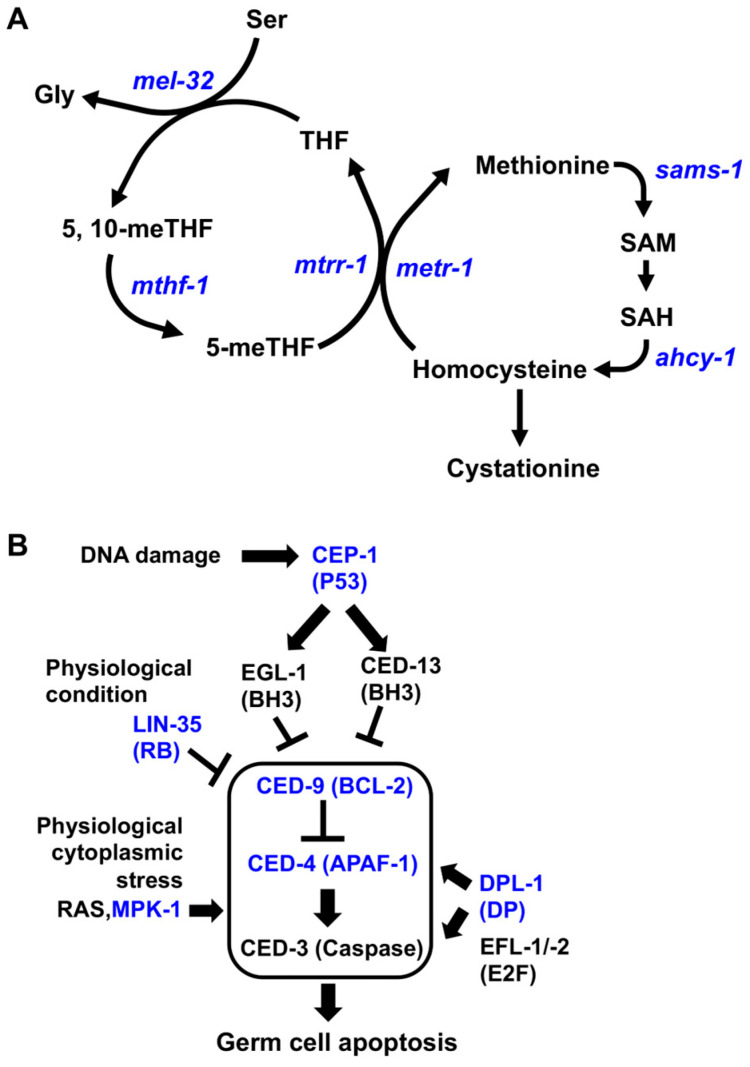
Methionine cycle and germ cell apoptosis pathway in *C. elegans*. (**A**) Schematic of the methionine cycle in *C. elegans*. THF, tetrahydrofolate; 5-meTHF, 5-methyltetrahydrofolate; 5,10-meTHF, 5,10-methyltetrahydrofolate; SAM, S-adenosyl methionine; SAH, S-adenosylhomocysteine. (**B**) Schematic of germ cell apoptosis pathway in *C. elegans*. The examined enzymes of the methionine cycle (**A**), core apoptotic genes (CED-9, CED-4, and CED-3), and its related genes (**B**) are indicated in blue. The scheme is modified from [17].

Methionine is necessary for cell growth and differentiation as an essential amino acid and functions as an initiator of protein synthesis across species [10]. It also plays major roles in metabolism, including the methionine cycle, transsulfuration pathway, and salvage cycle, which are well conserved from *Escherichia coli* to humans [10]. The first step in methionine metabolism is the methionine cycle, which is regulated by S-adenosylmethionine synthetase (*sams-1*), which leads to the biosynthesis of S-adenosylmethionine (SAM), a major methyl donor, from methionine (Figure 1A). The Met/SAM cycle is involved in the one-carbon metabolism and produces many important building blocks for the maintenance of the body [11]. Methionine deficiency induces renal apoptosis and cell cycle arrest [12]. It has also been reported that methionine supplementation improves reproduction, egg quality, antioxidant status, and the immune response [13]. Therefore, the balance of methionine composition in the body is important for maintaining healthy reproduction.

*C. elegans* has been used as the most powerful model organism to research the relationships between diet, metabolism, and reproduction [14,15]. Adult hermaphrodite gonads consist of two gonad arms, which are U-shaped and connected to the uterus. Approximately half of the germ cells die to support the development of oocytes, the so-called physiological germ cell apoptosis, during oogenesis at the adult stage [16,17]. Germ cell apoptosis is induced by the core apoptotic machinery, including anti-apoptotic protein CED-9/BCL-2 [16], caspase activator CED-4/APAF-1 [18], and the caspase CED-3/Caspase [18] (Figure 1B). The germ cell apoptosis physiologically induced by LIN-35/RB (tumor suppressor retinoblastoma) occurs in response to starvation stress, the RAS/MAPK signaling pathway, or E2F components [16,17,19]. Meanwhile, the induction of an increase in apoptosis in the germline via DNA damage is mediated by CEP-1, which is thought to be mammalian p53 [20] (Figure 1B). Germ cell apoptosis is important for maintaining germ cell health, oocyte quality, and offspring viability [21,22]. Thus, the question of how germ cell apoptosis is regulated by metabolic variation, specifically maternal diet, to maintain germ cell quality and offspring development was investigated in this study, using a caffeine-ingested *C. elegans* model.

## 2. Materials and Methods

### 2.1. C. elegans Strains and Culture

*C. elegans* strains were grown on nematode growth medium (NGM) agar plates seeded with *Escherichia coli* strain OP50 at 20 °C, as previously described [23]. To examine the effects of caffeine intake and methionine supplementation, either 10 mM caffeine (Sigma-Aldrich, St. Louis, MO, USA), which showed no toxicity for survival, or 10 mM of methionine (Sigma-Aldrich, St. Louis, MO, USA) was added to NGM, as previously described [24] (Supplementary Materials Figures S1 and S2). In this study, we used the strains as follows: N2 (*C. elegans* wild isolate, Bristol variety) and MD701: *bcIs39 [Plim-7::ced-1::gfp+lin-15(+)]* V to observe the engulfed apoptotic nuclei [20], MT3608: *unc-13(e51) ced-1(e1735)* I to count the germ cell corpses [25], DH1033: *bIs1 [vit-2::GFP+rol-6(su10060)]* X to measure the expression of vitellogenin [26].

### 2.2. Quantification of Methionine in CIA Using Gas Chromatography–Time-of-Flight Mass Spectrometry (GCTOF-MS)

Amino acids were extracted and analyzed as previously described [27], with three biological replicates. To perform GC-TOF-MS analysis, five thousand synchronized L4-stage wild-type animals were treated with or without caffeine for 24 h at 20 °C and analyzed as described previously [28,29].

### 2.3. Quantitative Reverse Transcriptase-Polymerase Chain Reaction (qRT-PCR)

The mRNA levels of the respective genes (Table 1) were measured using qRT-PCR, as previously described [24]. Briefly, caffeine-ingested adult hermaphrodites were collected in TRIzol. Oligo-dT primer and M-MLV reverse transcriptase were used for cDNA synthesis from the extracted total RNA. qPCR was performed using Power SYBR Green PCR Master Mix. The *act-1* mRNA was normalized for data analysis. Table 1 shows the primers used for qRT-PCR and the GenBank database accession number for *C. elegans*. 

### 2.4. Germ Cell Apoptosis Assay

Germ cell apoptosis was measured either using the reporter CED-1::GFP for engulfed apoptotic nuclei or by counting germ cell corpses, as previously described [30,31]. We used two methods to measure germ cell death for following reasons: (1) measuring the number of CED-1-positive germ cells using the CED-1::GFP transgenic animals to observe the engulfing cell during apoptosis and (2) measuring the number of cell corpses in *ced-1* mutant background, an engulfment defective mutant to check whether caffeine intake affects apoptosis through a defect in the engulfment pathway. The synchronized L4-stage animals were exposed to 10 mM of caffeine for 24 h at 20 °C and the adult hermaphrodites were prepared for live imaging with 10 mM levamisole in M9 buffer. The animals were mounted on a 4% agarose pad with a cover slip and images were captured using a fluorescence microscope (Zeiss Axioscope, Oberkochen, Germany). One gonad arm was measured per animal. Fifteen to twenty animals were examined in each condition.

### 2.5. Germ Cell Proliferation Assay

To observe the mitotic germ cells at the distal region of the gonad in CIA, we dissected the gonads and fixed them with cold methanol (100%) and cold acetone (100%) at −20 °C each for 10 min. The samples were immunostained with rabbit anti-phospho-histone H3 (1:500, EMD Millipore, 06-570, Burlington, MA, USA) and Alexa Fluor 594-conjugated goat anti-rabbit IgG (1:500, Invitrogen, A32740, Walthamma, MA, USA). Imaging was performed using a fluorescence microscope (Zeiss Axioscope, Oberkochen, Germany) and the number of PH3-positive germ cells was counted per gonad arm.

### 2.6. RNA Interference (RNAi) Assay

The soaking RNAi method was performed as described previously [32]. The dsRNAs of *cep-1*, *mpk-1*, *lin-35*, *dpl-1*, *sams-1*, *metr-1*, *mtrr-1*, and *vit-2* genes were synthesized *in vitro* using the respective cDNA template. The synchronized L4-stage animals were soaked in dsRNA solution for 24 h at 20 °C, transferred onto either caffeine-containing or caffeine-free NGM agar plates, and incubated for 24 h at 20 °C. Table 2 shows the primers used for the RNAi assay.

### 2.7. Western Blot Analysis

Approximately 100 adult animals for each condition, with three independent sets of experiments, were prepared and a total of 300 adult animals were used for whole-worm protein extraction for Western blotting. The ECL Western blotting detection kit (Amersham, Pittsburgh, PA, USA) was used for visualization and the LAS-300 (Fuji Film, Tokyo, Japan) was used for quantification. Table 3 shows the antibodies used in this study.

### 2.8. Observation of VIT-2::GFP Expression

Live animals in each condition were observed and imaged as previously described [24]. In brief, GFP-tagged fluorescent proteins were visualized in living worms by mounting them on 2% agarose pads with M9 buffer with 10 mM levamisole. Live fluorescent imaging was performed using a Zeiss microscope at 20× magnification. ImageJ software (Version 1.54c) was used for the quantification of fluorescence intensity.

### 2.9. Observation of Developmental Stages of Animals

To observe the growth rate of the offspring, the percentage of larval development was determined as the percentage of larvae of the total number of hatched embryos that reached each developmental stage, as previously described [33]. We observed the characteristics of each developmental stage, as shown in Table 4.

### 2.10. Statistical Analysis

GraphPad Prism 10.2.1 software was applied for statistical analyses and data graphs. The scatter dot plots with bars displays the standard deviation from the mean value. *n* values for each experiment are denoted in the corresponding figure legends. *p*-values were calculated using either Student’s *t*-test (between the two groups) or a one-way ANOVA test (between more than two groups) for the statistical evaluation of the data. We considered *p* values lower than 0.05 to be statistically significant.

## 3. Results 

### 3.1. Maternal Caffeine Intake Causes Alterations in Methionine Metabolism in C. elegans

**Figure 2:**
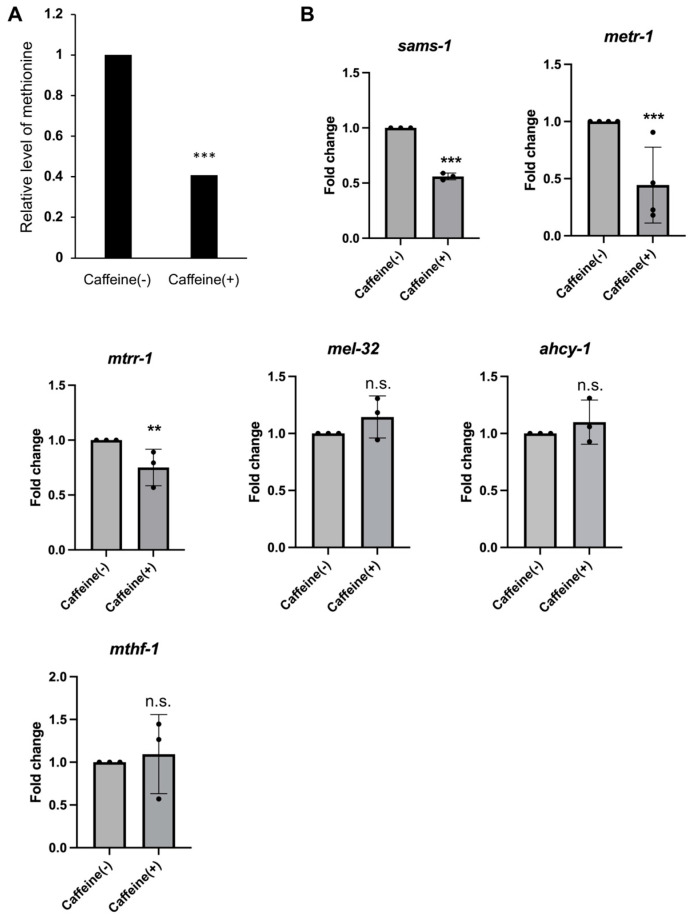
Caffeine intake caused alterations in methionine metabolism in *C. elegans*. (**A**) Fold change in methionine between the caffeine (−) and the caffeine (+) animals using gas chromatography. In total, 5000 adult animals were extracted to determine the methionine level for each group. *p* values were calculated using Student’s *t*-test. (**B**) Fold induction in mRNA of *sams-1*, *metr-1*, *mtrr-1*, *mel-32*, *ahcy-1*, and *mthf-1* in CIAs. *p* values were calculated using Student’s *t*-test. *** *p* < 0.001. ** *p* < 0.005. n.s., not significant. Error bars represent SD.

We previously showed that the decrease in reproduction and vitellogenesis caused by caffeine intake was improved by vitamin B12 supplementation, which acts as a co-factor in the Met/SAM cycle in *C. elegans* [34]. Considering the important role of methionine in cellular metabolism, we investigated its role in reproduction in caffeine-ingested animals (CIAs). We performed GC-TOF-MS chromatography to analyze the changes in methionine levels due to caffeine intake. The level of methionine in CIAs was significantly decreased by approximately 2-fold (0.408) compared to that in the caffeine (−) group (Figure 2A). This result indicates that caffeine intake causes a reduction in methionine levels in *C. elegans*. Given the decrease in methionine level due to caffeine intake, we investigated whether the methionine metabolic genes are affected by caffeine intake through measuring the mRNA level of *sams-1* (S-adenosyl methionine synthetase), *metr-1* (5-methyltetrahydrofolate-homocysteine methyltransferase (methionine synthase)), *mtrr-1* (methyltransferase reductase (methionine synthase reductase)), *mel-32* (serine hydroxymethyltransferase), *ahcy-1* (S-adenosylhomocysteine hydrolase), and *mthf-1* (methylenetetrahydrofolate reductase) genes in CIA using qRT-PCR (Figure 1A and Figure 2B). We found that the mRNA levels of *sams-1*, *metr-1*, and *mtrr-1* genes significantly decreased in CIAs, while the mRNA levels of *mel-32*, *ahcy-1*, and *mthf-1* genes were not significantly altered (Figure 1B). These results suggest that maternal caffeine intake causes a reduction in methionine levels by lowering the mRNA levels of *mtrr-1*, *metr-1*, and *sams-1* methionine cycle genes. 

### 3.2. Caffeine-Induced Impaired Methionine Cycle Increases Germ Cell Apoptosis in an LIN-35/RB-Dependent Manner

**Figure 3:**
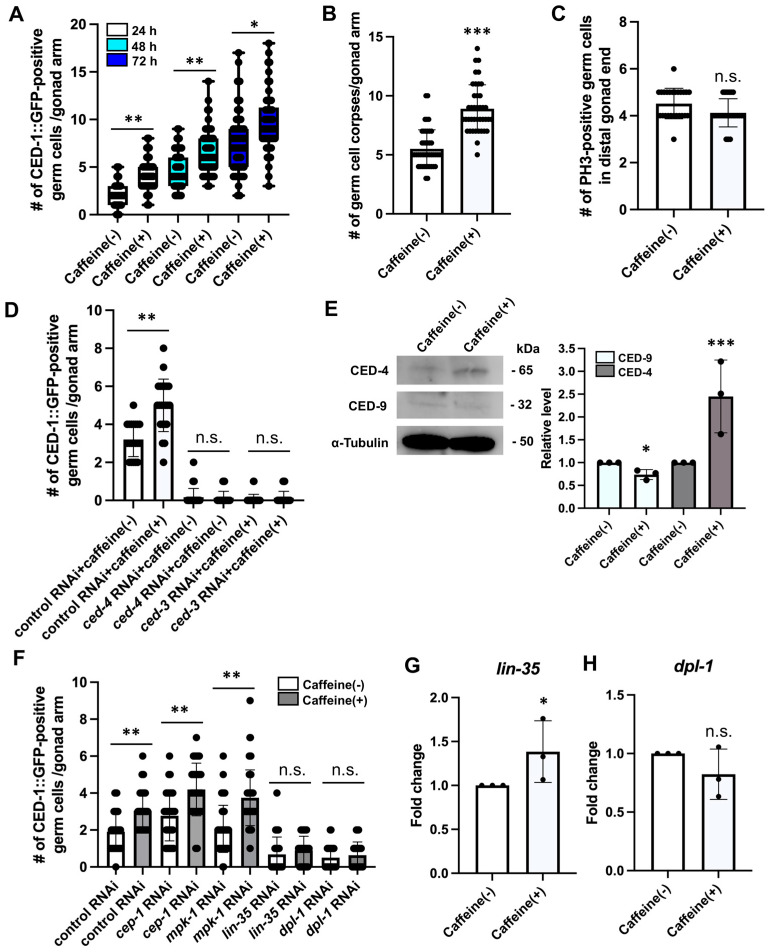
Caffeine intake increased germ cell apoptosis in an LIN-35/RB-dependent manner. (**A**) The number of apoptotic germ cells in MD701 transgenic animals treated with or without caffeine at 20 °C for 24 h, 48 h, and 72 h after the L4 stage. *n* > 30 in each condition. **, *p* < 0.005. *, *p* < 0.01 (one-way analysis of variance (ANOVA) with Tukey’s post hoc test). (**B**) The number of germ cell corpses in *ced-1*(*e1735*) mutants treated with or without caffeine at 20 °C for 24 h after the L4 stage. *n* > 30 in each condition. ***, *p* < 0.001 (Student’s *t*-test). (**C**) The number of PH3-positive germ cells in wild-type animals treated with or without caffeine at 20 °C for 24 h after the L4 stage. *n* > 30 in each condition. n.s., not significant (Student’s *t*-test). (**D**) The number of apoptotic germ cells by RNAi depletion of mock, *ced-4*, and *ced-3* in MD701 transgenic animals treated with or without caffeine at 20 °C for 24 h after the L4 stage. *n* > 30 in each condition. **, *p* < 0.005. n.s., not significant (ANOVA with Tukey’s post hoc test). (**E**) Western blot analysis of CED-4 and CED-9 in wild-type animals treated with or without caffeine at 20 °C for 24 h after the L4 stage. *n* > 300 in each condition. ***, *p* < 0.001. *, *p* < 0.01 (Student’s *t*-test). (**F**) The number of apoptotic germ cells by RNAi depletion of mock, *cep-1*, *mpk-1*, *lin-35*, and *dpl-1* in MD701 transgenic animals treated with or without caffeine at 20 °C for 24 h after the L4 stage. *n* > 30 in each condition. **, *p* < 0.005. n.s., not significant (ANOVA with Tukey’s post hoc test). (**G**,**H**) Fold induction in mRNA of *lin-35* (**G**) and *dpl-1* (**H**) in CIAs. *p* values were calculated using Student’s *t*-test. *, *p* < 0.01. n.s., not significant. Error bars represent SD.

Based on the decreased amount of methionine caused by caffeine intake, we investigated whether caffeine intake affects germ cell apoptosis or proliferation due to the caffeine-induced impairment of the methionine cycle. We observed the level of engulfment using the transgenic animals carrying the transgene *bcIs39 [Plim-7::ced-1::gfp]*, which expresses GFP-tagged CED-1 in gonadal sheath cells [31]. The level of engulfment was higher in CIAs than in the caffeine-free diet group and a noticeable increase was observed after 24 h of caffeine treatment (Figure 3A). We also scored the germ cell corpses in CIAs and confirmed that CIA gonads contained more germ cell corpses than the caffeine-free diet group at 24 h post-caffeine treatment (Figure 3B), indicating that caffeine intake increased the level of germ cell apoptosis, possibly through an altered methionine cycle. Next, we examined the effects of caffeine intake on germ cell proliferation. CIAs were immunostained with anti-PH3 (late G2 and m-phase markers) to examine mitotic germ cells. The number of PH3-positive germ cells in CIAs was not significantly altered compared with that in the control group (Figure 3C). These findings suggest that caffeine intake mainly affects germ cell apoptosis but not cell proliferation. To confirm that the increased level of germ cell apoptosis caused by caffeine intake was due to activation of the core apoptotic machinery, we performed *ced-4* and *ced-3* RNAi depletion. Caffeine treatment did not increase the number of apoptotic germ cells in the depletion of both *ced-4* and *ced-3* (Figure 3D). Western blot analysis was performed using anti-CED-4 and anti-CED-9 antibodies. CIAs contained higher levels of CED-4 and lower levels of CED-9 than the control group (Figure 3E). These results demonstrate that caffeine intake significantly increases germ cell apoptosis by inactivating CED-9, thereby activating CED-4, a core apoptotic machinery protein.

Next, we examined the pathways involved in caffeine-induced germ cell apoptosis. As shown in Figure 1B, the core apoptotic machinery is regulated by several pathways, including the activity of *cep-1* in response to DNA damage, *mpk-1* in response to physiological cytoplasmic stress, *lin-35* in response to physiological condition/starvation, and *dpl-1*, which regulates CED-4. Each pathway was examined using the RNAi depletion of each gene in CIAs. We found that germ cell apoptosis increased with the depletion of either *cep-1* or *mpk-1* RNAi (Figure 3F). In contrast, the RNAi depletion of *lin-35* and *dpl-1* did not result in a significant increase in germ cell apoptosis after caffeine intake (Figure 3F). These results suggest that *lin-35* and *dpl-1*, but not *cep-1* and *mpk-1*, are required to increase the germ cell apoptosis induced by caffeine intake. We further confirmed that the *lin-35* mRNA level, but not *dpl-1*, was increased by caffeine intake (Figure 3G,H). Our results indicate that caffeine intake increases germ cell apoptosis in an LIN-35/RB-dependent manner.

### 3.3. Methionine Metabolism Is Involved in Physiological Germ Cell Apoptosis and Vitellogenesis

**Figure 4:**
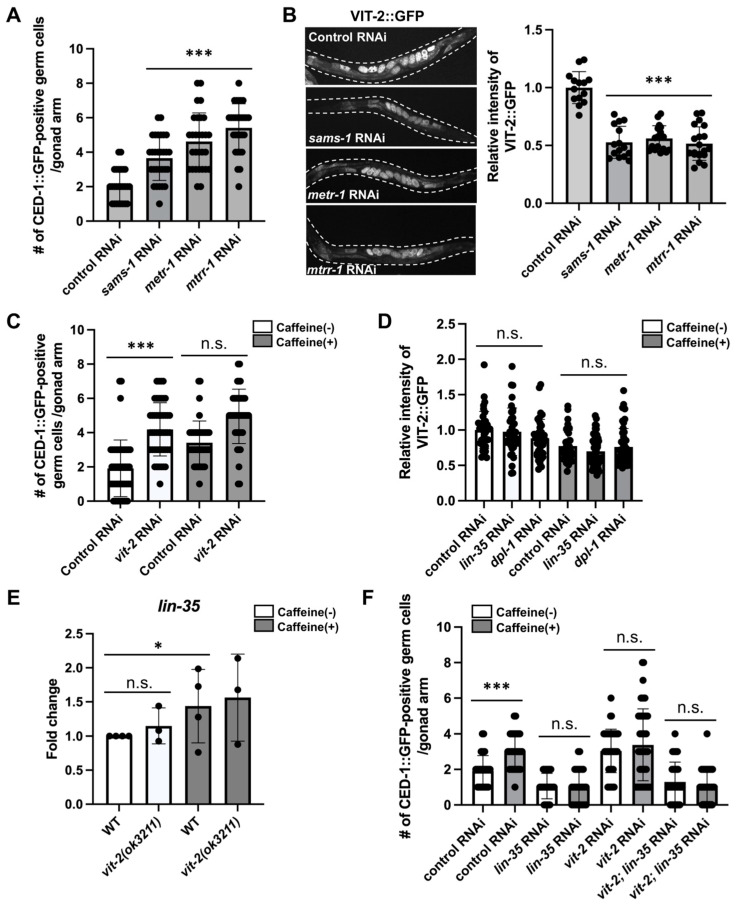
Methionine metabolism is involved in physiological germ cell apoptosis and vitellogenesis. (**A**) The number of apoptotic germ cells by RNAi depletion of mock, *sams-1*, *metr-1*, and *mtrr-1* in MD701 transgenic animals. *n* > 30 in each condition. *p* values were calculated using one-way analysis of variance (ANOVA) with Tukey’s post hoc test. ***, *p* < 0.001. Error bars represent SD. (**B**) VIT-2::GFP expression in DH1033 transgenic animals treated with mock, *sams-1*, *metr-1*, and *mtrr-1* RNAi. *n* > 30 in each condition. Relative intensity was examined using ImageJ analysis. *p* values were calculated using ANOVA with Tukey’s post hoc test. ***, *p* < 0.001. (**C**) The number of apoptotic germ cells by RNAi depletion of *vit-2* in CED-1::GFP transgenic animals with caffeine treatment. *n* > 30 in each condition. *p* values were calculated using ANOVA with Tukey’s post hoc test. ***, *p* < 0.001. n.s., not significant. Error bars represent SD. (**D**) VIT-2::GFP expression in DH1033 transgenic animals by RNAi depletion of *lin-35* and *dpl-1* with caffeine treatment. *n* > 30 in each condition. *p* values were calculated using ANOVA with Tukey’s post hoc test. n.s., not significant. Error bars represent SD. (**E**) Fold induction in mRNA of *lin-35* in N2 and *vit-2(ok3211)* mutants with caffeine treatment. *n* > 300 in each condition. *p* values were calculated using ANOVA with Tukey’s post hoc test. *, *p* < 0.01. n.s., not significant. Error bars represent SD. (**F**) The number of apoptotic germ cells by RNAi depletion of *lin-35* and *vit-2* in MD701 transgenic animals with caffeine treatment. *n* > 30 in each condition. ***, *p* < 0.001. n.s., not significant (ANOVA with Tukey’s post hoc test). Error bars represent SD.

Next, we confirmed whether the activity of genes essential for the methionine cycle affects germ cell apoptosis by RNAi depletion of *sams-1*, *metr-1*, and *mtrr-1*, which showed reduced mRNA transcript levels after caffeine intake (Figure 2B). We found that the RNAi depletion of *sams-1*, *metr-1*, or *mtrr-1* increased germ cell apoptosis (Figure 4A), suggesting that methionine metabolism is involved in physiological germ cell apoptosis. Next, we investigated whether vitellogenesis is affected by alterations in methionine metabolism in CIAs. We measured the level of vitellogenesis in transgenic animals carrying an integrated VIT-2::GFP transgene after the simultaneous depletion of *sams-1*, *metr-1*, or *mtrr-1* to suppress methionine metabolism. As reflected in the germ cell apoptosis phenotype due to impaired methionine metabolism, we observed that vitellogenesis was also significantly reduced by the RNAi depletion of *sams-1*, *metr-1*, and *mtrr-1* compared to the control group (Figure 4B). Furthermore, when CED-1::GFP transgenic animals with *vit-2* RNAi depletion were examined, the number of apoptotic germ cells significantly increased. However, the number of apoptotic germ cells in CIAs with *vit-2* RNAi depletion was comparable to that in the control group in CIAs (Figure 4C). These results suggest that vitellogenesis also affects physiological germ cell apoptosis and that caffeine intake and vitellogenesis share the same pathway to increase germ cell apoptosis.

In a previous study, caffeine intake reduces vitellogenesis [24]. We found that caffeine intake increases *lin-35* activity and the same pathway as vitellogenesis to induce germ cell apoptosis. To examine the mutual effects of vitellogenesis and *lin-35* activity on germ cell apoptosis induced by caffeine intake, we observed vitellogenesis expression with *lin-35* RNAi in CIAs. We found that the RNAi depletion of *lin-35* and *dpl-1* did not affect vitellogenesis in CIAs (Figure 4D). We also examined the *lin-35* activity in a *vit-2 (ok3211)* mutant background and found that this did not significantly alter the *lin-35* mRNA transcript level in the *vit-2* mutant compared to that in wild-type animals (Figure 4E). To elucidate the genetic interaction between *lin-35* and *vit-2* in the germ cell apoptosis pathway induced by caffeine intake, we performed double RNAi of *lin-35* and *vit-2* in CIAs and the control groups. Germ cell apoptosis induced by *vit-2* RNAi was abolished by *lin-35* RNAi depletion, suggesting that *vit-2* functions upstream of *lin-35* to negatively regulate the activity of *lin-35* (Figure 4F). *vit-2* and *lin-35* double RNAi showed a phenotype similar to that of *lin-35* single RNAi under both caffeine-free and caffeine-intake conditions (Figure 4F). Taken together, the decreased level of vitellogenesis in CIAs activates *lin-35* and, thus, increases germ cell apoptosis in CIAs (see Discussion).

### 3.4. Methionine Supplementation Alleviates the Effects of Caffeine Intake

**Figure 5:**
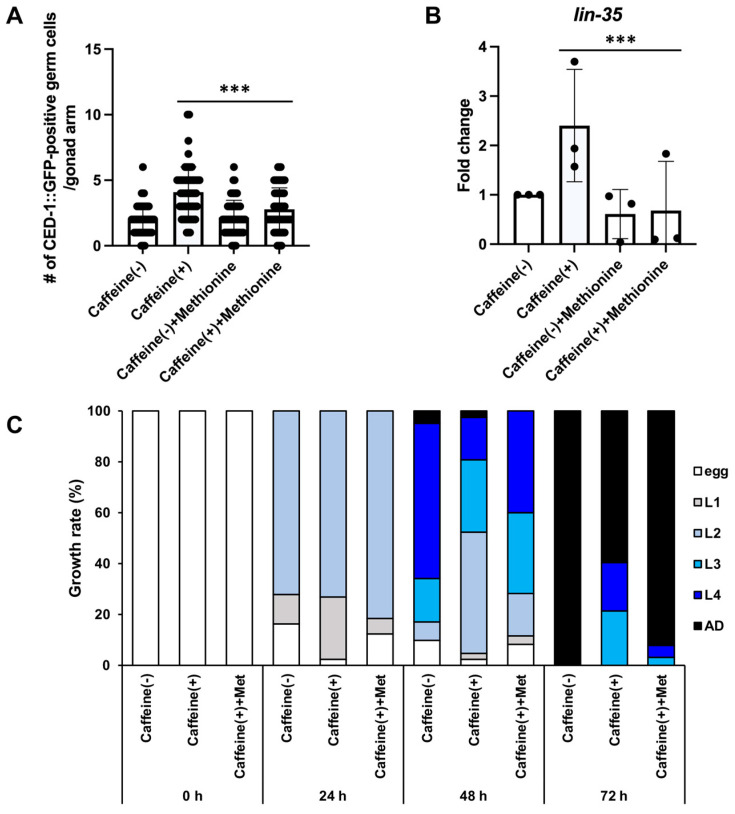
Methionine supplementation alleviates the effects of maternal caffeine intake. (**A**) The number of apoptotic germ cells in MD701 transgenic animals treated with caffeine and methionine. *n* > 30 in each condition. *p* values were calculated using one-way analysis of variance (ANOVA) with Tukey’s post hoc test. ***, *p* < 0.001. Error bars represent SD. (**B**) Fold induction in mRNA of *lin-35* in N2 with caffeine and methionine treatment. *n* > 300 in each condition. *p* values were calculated using ANOVA with Tukey’s post hoc test. ***, *p* < 0.001. Error bars represent SD. (**C**) Synchronized wild-type L4-stage mothers (P0) were treated with caffeine and methionine for 24 h. The embryos (F1) produced by each group of mothers were cultured at 20 °C and their developmental stages were measured until they reached adult stage. Each developmental stage in the F1 generation was determined by its size- and stage-specific morphological characteristics during development in caffeine-ingested P0 mother supplemented with methionine. *n* > 40 in each condition.

We found that the reduction in both reproduction and vitellogenesis by caffeine intake was improved by vitamin B12 supplementation, which is an important co-factor in methionine metabolism in a previous study. Considering these previous findings, we tested whether methionine supplementation plays a beneficial role in caffeine-induced germ cell apoptosis. We first tested whether methionine supplementation recovers the level of germ cell apoptosis in CIAs and found that the elevated level of germ cell apoptosis induced by caffeine intake was significantly reduced in CIAs with methionine supplementation (Figure 5A, Supplementary Materials Figure S3). We also confirmed that the increased level of *lin-35* mRNA transcript caused by caffeine intake was significantly decreased by methionine supplementation (Figure 5B), indicating that methionine supplementation plays a beneficial role in decreasing the level of germ cell apoptosis induced by caffeine intake. Maternal caffeine intake has been shown to retard offspring development by reducing vitellogenesis in *C. elegans* [24]. Therefore, we tested whether methionine supplementation could contribute to offspring growth in CIAs. We evaluated the subsequent F1 generation in CIAs with methionine supplementation. Interestingly, caffeine-fed mothers displayed a significant improvement in offspring developmental growth rate (approximately 59.5% adults with caffeine and 92.0% adults with caffeine + methionine supplementation at 72 h of growth). These results suggest that methionine supplementation has a beneficial effect on both caffeine-fed mothers and their subsequent generation. 

## 4. Discussion

**Figure 6:**
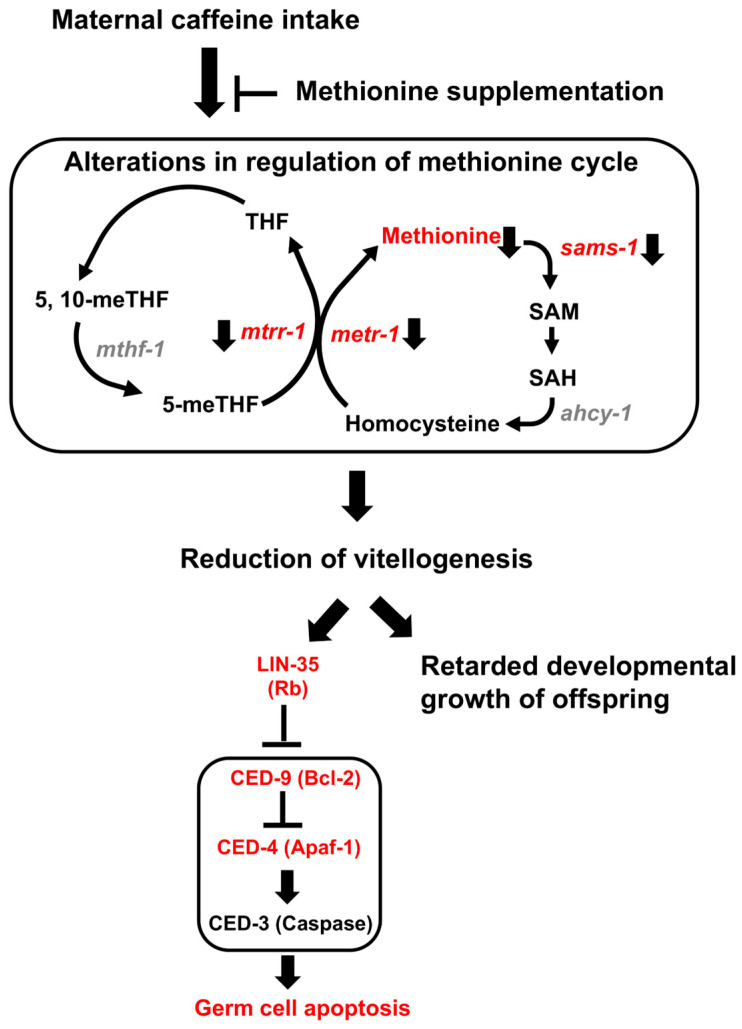
Working model. Maternal caffeine intake impairs the methionine cycle by reducing the level of methionine. This alteration decreases vitellogenesis and induces physiological germ cell apoptosis in an LIN-35/RB-dependent manner. When methionine is supplemented in CIAs, the level of vitellogenin and lin-35-depedent germ cell apoptosis recovers to normal levels. Additionally, delayed offspring growth due to maternal caffeine intake is also improved by methionine supplementation.

The previous study showed that maternal caffeine intake negatively affected offspring development in a *C. elegans* model [24]. In this study, we investigated the physiological alterations affecting germ cell health and offspring development following maternal caffeine intake. We found that maternal caffeine intake decreased methionine levels, which seemed to be due to the reduced expression of *sams-1*, *metr-1*, and *mtrr-1*, which are essential for the methionine cycle. The decreased amount of methionine resulted in a reduction in vitellogenesis, thus increasing the level of apoptosis in the germline and decreasing developmental growth in the offspring (Figure 6). Surprisingly, when methionine was supplemented in CIAs, the negative effects of maternal caffeine intake were alleviated. 

Germ cell apoptosis in *C. elegans* occurs during oogenesis at the adult stage to maintain oocyte quality so that the subsequent offspring development is normally proceeded [21,22]. A proper supply of yolk protein is essential for oocyte quality [24,26]. We previously found a reduction in vitellogenesis in CIAs [24]. Vitellogenesis in *C. elegans* at the adult stage is regulated by methionine supplementation [35]. Considering these findings, we propose that the reduction in vitellogenesis in CIAs is due to alterations in the methionine cycle. We found that caffeine intake significantly reduced methionine levels and vitellogenesis and, thus, increased germ cell apoptosis. Recently, it has been reported that maternal caffeine intake causes mitochondrial stress in the germ line of *C. elegans* [34], but the mechanism by which caffeine intake induced mitochondrial stress in the germline remains unclear. Interestingly, some studies have demonstrated an interrelationship between methionine metabolism and mitochondrial function with direct relevance [36,37,38]. Furthermore, the function of mitochondria is essential for maintaining oocyte quality [39,40,41]. Based on the previous findings and this study, it raises the possibility that maternal caffeine intake targets mitochondrial function by altering methionine metabolism, which induces an increased level of germ cell apoptosis during oogenesis. Hence, it is necessary to investigate the genetic interactions between mitochondrial stress response and methionine cycle genes during the germ cell apoptosis induced by maternal caffeine intake in future studies. 

The tumor suppressor retinoblastoma (RB) is a transcriptional regulator essential for normal cell cycle progression and apoptosis [42]. The RB is located in the outer mitochondrial membrane and promotes apoptosis [43,44,45]. It was reported that *lin-35*, a *C. elegans* homolog of *Rb*, is increased under starvation conditions to suppress the expression of *ced-9*, thereby holding open CED-4; then, it interacts with CED-3 to induce germ cell apoptosis [46]. Starvation conditions also promote *dpl-1*, which increases CED-4 accumulation [46]. In this study, we showed that maternal caffeine intake increased germ cell apoptosis in an LIN-35/RB-dependent manner. Although maternal caffeine intake appears to partially require DPL-1 activity, germ cell apoptosis induced by caffeine intake appears to be distinct from increased germ cell apoptosis induced by starvation. There is a distinct difference between starvation and caffeine-induced germ cell apoptosis, in that starvation caused an increase in the expression of both *lin-35* and *dpl-1*, whereas caffeine intake exclusively increased the *lin-35* mRNA level (Figure 3H). This suggests that the increased germ cell apoptosis resulting from caffeine intake is regulated somewhat differently from the starvation-induced LIN-35 pathway. There are intriguing questions thar remain to be answered, as follows: how does maternal caffeine intake induce LIN-35 activity, how do alterations in the methionine cycle and vitellogenesis regulate LIN-35 activity, and, in particular, the possibility that *C. elegans* LIN-35 functions in mitochondria to promote apoptosis in the germ line?

Maternal nutritional status is considered a critical factor in the interaction with major metabolic pathways, shaping both health and individual phenotypes during fetal development [47,48,49]. Methionine is an essential methyl donor for DNA methylation, which affects gene expression by epigenetic alterations and is linked to the offspring transcriptome, which is mediated by changes in DNA methylation [50]. Therefore, maternal methionine metabolism is important for embryonic development, growth, and offspring health. We found that impaired methionine metabolism caused by maternal caffeine intake reduced vitellogenesis. Interestingly, the decrease in vitellogenesis due to caffeine intake caused an upregulation in germ cell apoptosis; however, unlike the increased *lin-35* due to caffeine intake in the wild-type background, there was no change in *lin-35* mRNA level in the *vit-2* mutant (Figure 4E). This may be due to redundancy, as the *vit-2* single mutation is not sufficient to increase the mRNA level of *lin-35*, and in *C. elegans* there are six orthologous genes from *vit-1* to *vit-6* for vitellogenesis [26]. Considering the beneficial effect of methionine on egg and milk production reported in previous studies [51,52], it is thought to play a specific role in nutrient metabolism and reproduction. When methionine was supplemented in CIAs, both reduced vitellogenesis and the retarded developmental growth of offspring caused by caffeine intake were improved, consistent with its beneficial role in reproduction. 

*C. elegans* is popular as an excellent animal model to investigate chemical toxicology. We showed that maternal caffeine intake led to the alterations in methionine metabolism and germ cell apoptosis in *C. elegans*. However, there are limitations in directly applying it to human physiology. Moreover, the delivery methods for chemicals in *C. elegans* are indirect and can vary [53,54,55,56] and the cuticle of *C. elegans* also hinders the uptake of chemicals. Therefore, the concentration of caffeine treatment in this study using *C. elegans* may be at a low level.

Collectively, these results indicate that methionine alleviated the harmful effects of maternal caffeine consumption. Our study suggests new insights into the negative effects of maternal metabolic alterations on reproductive and offspring health.

## 5. Conclusions

This study showed that maternal caffeine intake decreased both methionine levels and expression levels of genes for essential enzymes modulating methionine cycle, such as *sams-1*, *metr-1*, *mtrr-1*. We also found that the caffeine-induced impairment of methionine metabolism decreased vitellogenesis and increased germ cell apoptosis in an LIN-35/RB-dependent manner. Methionine supplementation in caffeine-ingested animals alleviated caffeine-induced negative effects on reproduction. Our findings suggest that methionine supplementation has a beneficial effect on germ cell health and offspring development through the regulation of vitellogenesis.

## Figures and Tables

**Table 1:** The primers used for qRT-PCR and the GenBank database accession number for *C. elegans* mRNA.

mRNA Target		Primer Sequence	GenBank Database
*act-1*	forward	5’-CCAGGAATTGCTGATCGTATGCAGAA-3′	NC_003283.11, Chr V: 11081052.11082415: 133 bp
	reverse	5’-TGGAGAGGGAAGCGAGGATAG-3′	
*sams-1*	forward	5′-ATTATCAAGGAGCTCGACCT-3′	NC_003284.9, Chr X: 11965962.11969893: 318 bp
	reverse	5′-ATGGGAACTCAGAGTGACC-3′	
*metr-1*	forward	5′-GGAGCAGCTACTGGTAGAC-3′	NC_003280.10, Chr II: 10926561.10931078: 194 bp
	reverse	5′-CACAGATGGCGAAATTGAGAG-3′	
*mtrr-1*	forward	5′-TACGTTCTTCTCGGTCTCG-3′	NC_003280.10, Chr II: 9285975.9289132: 121 bp
	reverse	5′-AGAGCTGTCAGTTGTTTGTC-3′	
*mel-32*	forward	5′-TGACTCATGGATTCTTCACCC-3′	NC_003281.10, Chr III: 6440148.6442815, complement: 119 bp
	reverse	5′-GATCAACCTTGTATGGAAGAGAC-3′	
*ahcy-1*	forward	5′-CGATTGCGAGATTGACGTC-3′	NC_003279.8, Chr I: 6836469.6838140: 221 bp
	reverse	5′-GTGTAACGGTCAACCTGTG-3′	
*mthf-1*	forward	5′-GTTGAGACCGATGAGAATGC-3	NC_003280.10, Chr II: 7773908.7777823, complement: 306 bp
	reverse	5′-TTCATAATGCTTTGGTGACCAG-3′	
*lin-35*	forward	5′-ACTGGAATTCCGTCCACTTG-3′	NC_003279.8, Chr I: 5807709.5815637: 327 bp
	reverse	5′-TCCGCTCATCAATACTTCCA-3′	
*dpl-1*	forward	5′-GGTCAGAGATATAGAGTTCGTCC-3′	NC_003280.10, Chr II: 9169910.9172565: 402 bp
	reverse	5′-CTGATGTCCAGAAATGCTTCCAG-3′.	

**Table 2:** The primers used for the RNAi assay.

Primer	Primer Sequence
T7 primer	5′-GTAATACGACTCACTATAGGGC-3′
CMo422 primer	5′-GCGTAATACGACTCACTATAGGGAACAAAAGCTGGAGCT-3′

**Table 3:** The antibodies used for this study.

Primary Antibodies				
Antigen	Host	Reference		Dilution
CED-9	Rabbit	Santa Cruz Biotechnology, Dallas, TX, USA		1:500
CED-4	Goat	Santa Cruz Biotechnology, Dallas, TX, USA		1:100
α-tubulin	Mouse	Sigma-Aldrich, St. Louis, MO, USA		1:200
**Secondary Antibodies**				
**Antigen**	**Host**	**Reference**	**Conjugated**	**Dilution**
Anti-rabbit IgG	Goat	Santa Cruz Biotechnology, Dallas, TX, USA	HRP	1:10,000
Anti-goat IgG	Donkey	Santa Cruz Biotechnology, Dallas, TX, USA	HRP	1:10,000
Anti-mouse IgG	Donkey	Jackson ImmunoResearch, PA, USA	HRP	1:1000

**Table 4:** Characteristics of each developmental stage in *C. elegans*.

Developmental Stages	Characteristics
L1 stage	smallest larvae less than 0.3 mm
L2 stage	larva larger than L1 but have no characteristics of L3 (body length, 0.3–0.4 mm)
L3 stage	larvae containing a white spot at the vulva region (body length, 0.4–0.6 mm)
L4 stage	larvae containing a half-moon-like shape at the vulva region (body length, 0.6–0.8 mm)
Adult stage	worms with an opened vulva with eggs in the uterus

## Data Availability

Data are contained within the article and Supplementary Materials.

## Supplementary Materials

The following supporting information can be downloaded at: https://www.mdpi.com/article/10.3390/nu16060894/s1, Figure S1: Experimental schemes for caffeine treatment and methionine supplementation in *C. elegans*; Figure S2: Survival assay of *C. elegans* treated with caffeine; and Figure S3: Vitellogenin (VIT-2::GFP) expression by caffeine treatment.

## Abbreviations

Abbreviation	Definition
*mel-32*	Serine hydroxymethyltransferase
*mthf-1*	Methylenetetrahydrofolate reductase (NADPH)
*mtrr-1*	Methionine synthase reductase
*sams-1*	S-adenosylmethionine synthase
*metr-1*	5-methyltetrahydrofolate-homocysteine methyltransferase
*ahcy-1*	Adenosylhomocysteinase
*cep-1*	*C. elegans* p-53-like protein
p53	Tumor protein
*lin-35*	Retinoblastoma-like protein homolog
RB	Retinoblastoma-associated protein
*ced-9*	Apoptosis regulator
Bcl-2	BCL2 apoptosis regulator
*ced-4*	Cell death protein 4
Apaf-1	Apoptotic peptidase activating factor 1
*ced-3*	Cell death protein 3 subunit p17
Caspase	Caspase-3
*dpl-1*	Vertebrate transcription factor DP-like
DP	DP transcription factor
*vit-2*	Vitellogenin structural genes (yolk protein genes)
*ced-1*	Cell death abnormality protein 1
