# Supplementary material for: Methionine Supplementation Alleviates the Germ Cell Apoptosis Increased by Maternal Caffeine Intake in a C. elegans Model
Source: Nutrients. 2024 Mar 20;16(6):894. doi: 10.3390/nu16060894 (PMC10974396; doi:10.3390/nu16060894)
Supplement: Supplementary file 1 [file nutrients-16-00894-s001.zip › nutrients-2909502-supplementary.pdf]

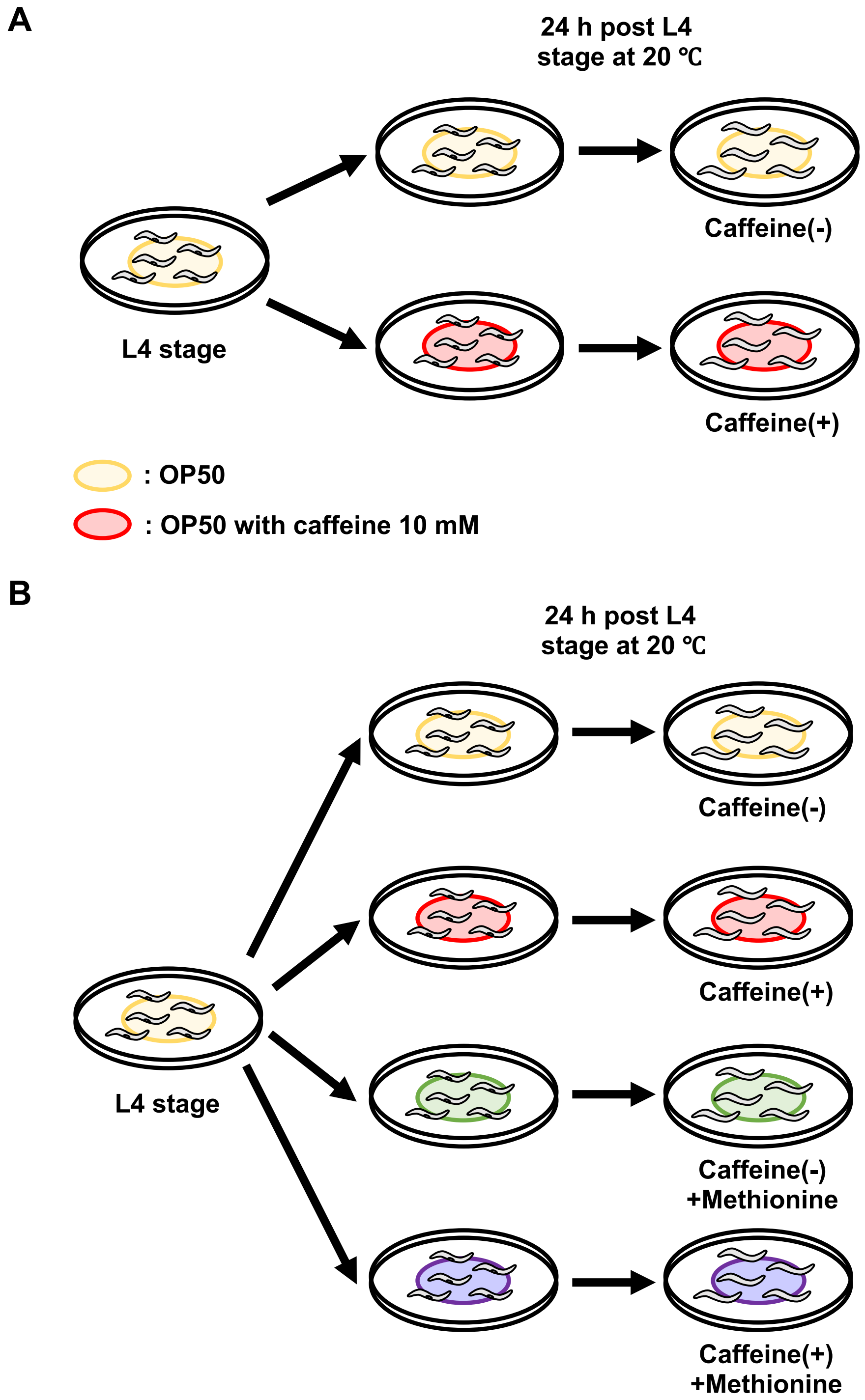

Figure S1. Experimental schemes for caffeine treatment and methionine supplementation in *C. elegans*. Experimental schemes for caffeine treatment and methionine supplementation in *C. elegans*. (A) Experimental scheme of caffeine treatment. 10 mM of caffeine was added to NGM plate to observe the effect of caffeine. L4 stage-worms were transferred to NGM plates with or without caffeine at 20 ℃ for 24 h. (B) Experimental scheme of caffeine + methionine supplementation. 10 mM of caffeine and 10 mM of methionine were added to NGM plate to observe the effect methionine with caffeine treatment. L4 stage-mothers were exposed to NGM plates with or without caffeine and methionine at 20 ℃ for 24 h .

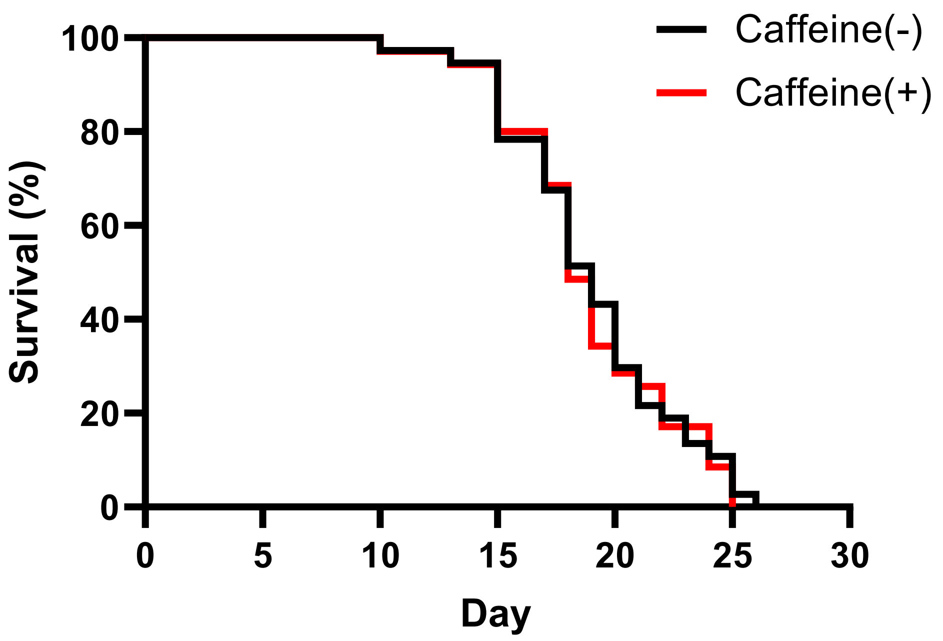

Figure S2. Survival assay of *C. elegans* treated with caffeine. The survival curves of wild-type N2 treated with 10 mM of caffeine. L4 stage-worms maintained at 20°C and live worms were counted during transfer to freshly made OP50 seeded NGM agar plates with or without caffeine every 2-3 days until death. n > 40 in each condition.

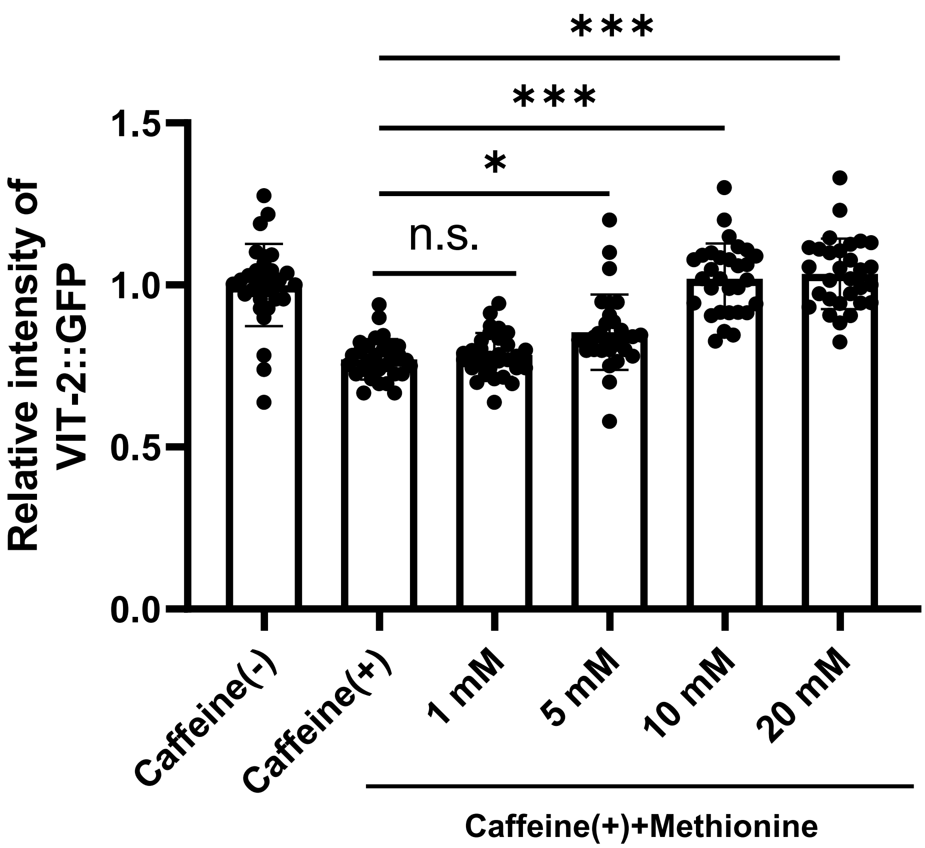

Figure S3. Vitellogenin (VIT-2::GFP) expression by caffeine treatment. The synchronized L4 stage of VIT-2::GFP transgenic animals were treated with 0 to 20 mM of methionine with 10 mM caffeine at 20℃ for 24 h. $n > 40$ in each condition. P value was calculated using one-way analysis of variance (ANOVA) with Tukey's post hoc test. ***, $p < 0.001$. *, $p < 0.01$. n.s., not significant. Error bars represent SD.
